# Retrospective review of factors associated with severe hospitalised community‐acquired influenza in a tertiary paediatric hospital in South Australia

**DOI:** 10.1111/irv.12403

**Published:** 2016-08-08

**Authors:** Nerissa Lakhan, Michelle Clarke, Suja M. Mathew, Helen Marshall

**Affiliations:** ^1^ Vaccinology and Immunology Research Trials UnitWomen's and Children's HospitalAdelaideSAAustralia; ^2^ School of MedicineUniversity of AdelaideAdelaideSAAustralia; ^3^ Flinders University and Flinders Medical CentreAdelaideSAAustralia; ^4^ School of Public HealthUniversity of AdelaideAdelaideSAAustralia; ^5^ Robinson Research InstituteUniversity of AdelaideAdelaideSAAustralia

**Keywords:** children, disease burden, hospitalisation, influenza

## Abstract

**Background:**

Influenza infection can result in severe disease with debilitating complications. Young children have the highest rate of influenza hospitalisations with various factors influencing influenza susceptibility and severity.

**Objectives:**

This study aimed to determine the disease burden and assess risk factors for severe hospitalised influenza in South Australian children under 5 years of age.

**Methods:**

Influenza admissions to the tertiary paediatric hospital in South Australia from 2008 to 2012 were identified. Data from laboratory‐confirmed influenza cases were collected, including infecting influenza strain, co‐infections, prematurity, pre‐existing medical comorbidities and other potential risk factors. Predictors of high‐level care were assessed using logistic regression.

**Results:**

A total of 267 children with laboratory‐confirmed influenza were hospitalised. Of these, 147 admissions (53%) occurred in children without underlying medical risk factors. Eighteen children (7%) required high‐level care, of which 11 (61%) had no underlying medical risk factors. No deaths were reported. The majority of children were unimmunised against influenza. Co‐infections were identified in 40% of children (*n* = 107). Influenza B was associated with a requirement for higher care (OR 3.7, CI 1.3–10.9, *P *= .02) as was a history of food allergies (OR 9.7, CI 1.5–61.4, *P *= .02) and iron deficiency anaemia (OR 4.8, CI 1.4–16.1, *P *= .01).

**Conclusions:**

Influenza can be a severe illness, even in children without underlying medical conditions. The identification of Influenza B strain, history of food allergies and iron deficiency anaemia as predictors of severity in hospitalised cases warrants further investigation and may have important implications for preventative strategies to reduce the burden of childhood influenza.

## 1 Introduction

Influenza is a highly contagious infection that can result in debilitating complications. There are many factors which may influence the susceptibility and severity of disease and disease outcomes. These include the influenza strain and subtype,^1^, ^2^, ^3^ current methods of treatment and prevention as well as factors associated with the infected individual such as age,^4^ the presence of co‐infections and comorbidities.^5^ Underlying chronic medical conditions including cardiac disease, neurological conditions,^2^ impaired immunity,^6^ obesity^5^ and chronic respiratory conditions including asthma^3^ may affect the severity of influenza infection in children. Many previous papers have identified risk factors during the H1N1 Influenza pandemic, with less known about predictors of severe disease during other non‐pandemic influenza seasons.

Children under 5 years of age have increased susceptibility to becoming infected with influenza and are at risk of more severe disease.^7^ They have the highest rate of influenza notifications compared with all other age groups and the highest hospitalisation rates.^8^ In 2012, there were 44 571 cases of laboratory‐confirmed influenza in Australia with 6286 of these cases reported in South Australia.^9^ The proportion of influenza notifications occurring in children aged less than 5 years between 2008 and 2014 in Australia varied between 10% (in 2009) and 15% (2008 and 2012). Children are the major viral reservoir for influenza, spreading it to their families and the community.^10^, ^11^ A US study of childhood mortality from seasonal influenza indicated that the majority of childhood deaths attributed to influenza occur in those less than 5 years of age.^12^

Influenza infection can range from asymptomatic infection to causing a spectrum of respiratory problems and complications, which can vary between types and subtypes of circulating influenza. The predominant circulating influenza types and strains in Australia varied during 2008–2014. According to the Australian influenza surveillance reports, in 2008, there was a predominance of Influenza B and A(H3N2),^13^ a pandemic of influenza A(H1N1) in 2009,^14^ the same also predominating during 2010–11.^15^, ^16^ In 2012–13, the predominant circulating influenza types were A(H3N2) and influenza B.^17^ A higher proportion of influenza B was reported in 2013 than in previous years. In 2014, influenza A(H1N1) was the predominant type in all but two states (New South Wales, Australian Capital Territory) where H3N2 predominated.

The aims of this study were to determine the burden of laboratory‐confirmed influenza requiring hospitalisation in children under 5 years of age in South Australia from 2008 to 2012 and investigate factors associated with more severe disease (defined as requiring intensive or high dependency care). In particular, the study aimed to identify the different types and strains of influenza virus causing disease in hospitalised children to assess the impact of strain variation and known and potential risk factors such as pre‐existing comorbidities, co‐infections and prematurity on severity of disease.

## 2 Materials and Methods

We performed a retrospective observational study of children under 5 years of age who were admitted with a diagnosis of influenza to the tertiary paediatric hospital in South Australia, between January 2008 and December 2012. We used ICD‐10‐AM (International classification of diseases, version 10, Australian modified) codes J09, J10 and J11 to identify potential hospital admissions (admission for influenza due to a certain identified influenza virus, influenza due to other identified influenza virus and influenza with an unidentified virus). Eligible cases required confirmed laboratory evidence of influenza (e.g. polymerase chain reaction or positive culture) as per Australian Government Department of Health and Ageing case definitions for confirmed influenza infection. Nosocomial cases were captured but excluded from analysis as disease severity may be different. Nosocomial cases were defined as patients admitted to hospital for other primary medical reasons who subsequently contracted an influenza infection during hospital stay (no symptoms of influenza at time of admission and influenza diagnosis >48 hours following admission). The study was reviewed and approved by the Women's and Children's Health Network Human Research Ethics Committee.

### 2.1 Data Collection

Data from hospital admissions were collected using a standardised questionnaire. Data included patient demographics, clinical symptoms, management and laboratory findings including influenza type and strain. Data included in the data collection form were based on review of the literature and potential clinical factors identified by clinicians. Strains were either typed as influenza A or Influenza B, with influenza A strains further subtyped as either H1N1 or not H1N1 as per SA Pathology laboratory practice from 2009 to 2012. Influenza immunisation history, where available, was sourced from the medical records or the Australian Childhood Immunisation Register. Fully vaccinated was defined as either two doses of Trivalent Influenza Vaccine (TIV) at least 21 days apart and at least 2 weeks prior to presentation or one dose at least 2 weeks prior to presentation and receipt of at least one TIV dose in a previous year. Unvaccinated children were those not receiving TIV in the year of or prior to admission or receiving the vaccine less than 2 weeks prior to presentation. Definitions for laboratory results above or below normal range were based on normal ranges for age.

### 2.2 Data processing and statistical analysis

Data were analysed using STATA^®^ IC11. Weight for age *Z*‐Scores were calculated using EpiInfo^™^ 3.5.4. Children with a weight for age Z‐score ≥95% percentile were considered obese for analysis as per Australian clinical practice guidelines.^18^

Hospitalised influenza cases were classified as severe if they required high‐level care (ICU/High Dependency Unit (HDU) admissions) and were compared with those that did not (short‐stay ward or general admission). Associations with severity were investigated with logistic regression. *P* values <.05 were considered significant. Results were reported as odds ratios (OR) with 95% confidence intervals.

Univariate and multivariable logistic regression was used to determine which factors were associated with severe disease. Multivariable models included all variables that had complete data (>80%) and *P* values <.1 on univariate regression analyses for associations with severity. A restrictive *P* value of ≤.1 was chosen due to the small number of children who were classified as severe (*n* = 18). In addition, we included age category (<2 or ≥2 years of age) and pre‐existing risk factors (asthma, cystic fibrosis, chronic lung disease, heart disease, diabetes, chronic metabolic disease, kidney disease and failure, immunocompromised, neurological disorder and blood disorder as identified in the Australian Immunisation Handbook^19^) in the multivariable model, based on their clinical relevance and potential for confounding.

## 3 Results

A coding review identified 510 influenza admissions in children less than 5 years of age, of which 279 were laboratory‐confirmed cases. Of these, 267 admissions were included in the analysis and 12 excluded as they were hospital‐acquired (nosocomial) influenza cases. Almost all of the influenza cases (260/267, 97.4%) were confirmed by PCR. There were no reported deaths among the 267 cases.

### 3.1 Admissions due to influenza

**Figure 1:**
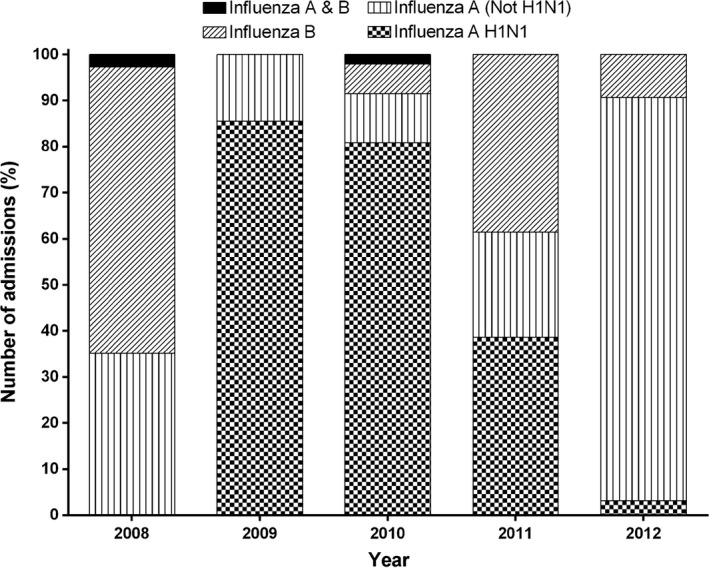
Influenza subtypes/lineages in children hospitalised with influenza under 5 years of age from 2008 to 2012. *n* = 267

The number of influenza admissions per year was highest in 2012 (*n* = 64) and lowest in 2008 (*n* = 37) (Table 1). Most admissions, (61%), were in children less than 2 years of age. Fifty‐six per cent (149/267) of hospitalised cases were male and 9.4% (25/267) were Aboriginal or Torres Strait Islander children. Forty‐three per cent (115/267) of admissions over the 5‐year period were due to influenza A H1N1 and 36% (96/267) were due to influenza A non‐H1N1 strains, (presumably mostly A(H3N2). A further 20% (54/267) were due to influenza B, and in two cases, both influenza A and B were identified (Fig. 1).

**Table 1:** General demographics of cohort and admission characteristics; *n* = 267

Variable	Level	Number	Percentage
Gender	Male	149	55.8
	Female	118	44.2
Ethnicity	Aboriginal/Torres Strait Islander	25	9.4
	Other	242	90.6
Country of birth	Australia	258	96.6
	Other	9	3.4
Age (y)	<1	86	32.2
	1–<2	78	29.2
	2–<3	32	12.0
	3–<4	39	14.6
	4–<5	32	12.0
Year of Admission	2008	37	13.8
	2009	62	23.2
	2010	47	17.6
	2011	57	21.4
	2012	64	24.0
Type of admission	HDU/ICU	18	6.7
	General admission or short stay	249	93.3
Duration of stay	0–<2 d	114	42.7
	2–6 d	120	44.9
	7‐ More days	33	12.4
Influenza strains (total)	Influenza A (H1N1)	115	43.1
	Influenza A (non‐H1N1)	96	36.0
	Influenza B	54	20.2
	Influenza A+B	2	0.7
Influenza strains by year 2008(37 cases)	Influenza A (H1N1)	0	0.0
	Influenza A (non‐H1N1)	13	35.1
	Influenza B	23	62.2
	Influenza A+B	1	2.7
2009(62 cases)	Influenza A (H1N1)	53	85.5
	Influenza A (non‐H1N1)	9	14.5
	Influenza B	0	0.0
	Influenza A+B	0	0.0
2010(47 cases)	Influenza A (H1N1)	38	80.8
	Influenza A (non‐H1N1)	5	10.6
	Influenza B	3	6.4
	Influenza A+B	1	2.1
2011(57 cases)	Influenza A (H1N1)	22	38.6
	Influenza A (non‐H1N1)	13	22.8
	Influenza B	22	38.6
	Influenza A+B	0	0.0
2012(64 cases)	Influenza A (H1N1)	2	3.1
	Influenza A (non‐H1N1)	56	87.5
	Influenza B	6	9.4
	Influenza A+B	0	0.0

The most common presenting symptoms for children hospitalised with influenza were cough, fever, rhinorrhoea and loss of appetite, which were present in over 50% of children. Febrile convulsions occurred in approximately 15% of children and pneumonia in 24% (*n* = 64/267). No cases of Guillain–Barré syndrome were recorded.

From our eligible cohort, 7% (18/267) of children required high‐level care (Table 2). Forty per cent (107/267) of admissions required oxygen therapy and 53% (139/264) required fluid replacement. Medication usage during admission was available for the majority of cases (*n* = 264, 99%). Ninety‐two per cent (242/264) of children were administered paracetamol and 31% (81/264) were administered non‐steroidal anti‐inflammatory drugs. Forty‐nine per cent (130/264) were administered antibiotics and nearly 14% (36/264) were given the antiviral, Oseltamivir. An elevated white cell count and C‐reactive protein was seen in the majority of those tested (56%, 108/195) and (70%, 99/142), respectively.

**Table 2:** Characteristics of the cases that required high‐level care (*n* = 18)

	Did not require ICU(*n* = 249)	Required ICU (severe)(*n* = 18)	*P* b value
	N (%)	N (%)	
Any medical risk factor a	115 (46.2)	11 (61.1)	.22
Influenza B strain	47 (19.0)	7 (38.9)	.04
Iron‐deficient anaemia	21 (8.4)	5 (27.8)	.01
Food allergy	6 (2.4)	2 (11.1)	.09
Proportion less than 2 y of age	150 (60.2)	14 (77.8)	.14
Antiviral prescribed	34 (13.7)	2 (11.8)	.82
Antibiotic prescribed	121 (48.8)	9 (52.9)	.74
Co‐infection identified	99 (39.8)	8 (47.1)	.55

a As defined in AIH.^14^

b *P* value is chi‐square or Fisher's exact as appropriate

Fifty‐three per cent (141/267) of children admitted to hospital with confirmed influenza did not have any of the medical risk factors for severe influenza as identified in the Australian Immunisation Handbook.^19^

### 3.2 Nosocomial cases

There were 12 nosocomial cases from the 279 admissions, which occurred in all age groups but predominantly in those under 1 year of age. The predominant strain identified was A(H1N1). Eight of the 12 nosocomial cases required ICU/HDU care. Eight were born prematurely (<37 weeks). The majority of nosocomial cases (8/12) had medical comorbidities and 7 of 12 had co‐infections with other bacterial or viral pathogens.

### 3.3 Previously Immunised children

From the 267 influenza admissions, only four children had documented evidence of having received an influenza vaccination in the year prior to admission. Two of these children were hospitalised in 2011, one with influenza A(H1N1) and one with influenza B. The other two children were admitted in 2012, both with influenza A non‐H1N1 strains. Two of the vaccinated children admitted with influenza had underlying comorbidities. None of the vaccinated children required ICU management.

### 3.4 Complications and Co‐infections

The presence of a co‐infection was identified in 40% (107/267) of children hospitalised with influenza. Eighty‐six per cent (92/107) of the co‐infections were due to other viruses (Parainfluenza 1 & 3, human metapneumovirus, respiratory syncytial virus, hepatitis, adenovirus, rhinovirus and norovirus), 24% (26/107) were due to bacteria (Staphylococcus aureus, Moraxella catarrhalis, Haemophilis influenza, Streptococcus, Neisseria meningitides, Pseudomonas aeruginosa, Escherichia coli, Salmonella, Pertussis, Mycoplasma) and 10% (11/107) were due to both bacteria and viruses. Seventy‐four per cent (198/267) of children hospitalised with influenza had complications, including pneumonia in 24% (64/267) and bronchiolitis in 16% (43/267).

### 3.5 Comorbidities and Potential Risk Factors

Comorbidities were analysed as potential predictors of severity in a univariate analysis (Table 3). Iron deficiency anaemia was reported in 6.7% of children hospitalised with influenza and was significantly associated with a higher risk of more severe influenza infection (OR 4.2, 95% CI 1.4–12.9; *P *= .01). Influenza B was associated with an increased risk of severe disease (OR 2.7, 95% CI 1.0–7.4; *P *= .05) compared with influenza A strains. Although not statistically significant, a history of food allergy showed a trend with increased odds of more severe disease (OR 5.1, 95% CI 1.0–27.1; *P *= .06).

**Table 3:** Risk factors for severe influenza and univariate analysis; *n* = 267

Variable	Level	N	Total N for variable	%	Odds ratio	Lower95% CI	Upper95% CI	*P*‐value
Prematurity	Yes	45	180	25.0	2.3	0.7	7.7	.17
Birthweight	<2.5 kg	25	130	19.2	2.7	0.6	12.3	.19
Gender	Male	149	267	55.8	1.3	0.5	3.4	.64
	Female	118		44.2	1.0			
Age	<2 y old	164	267	61.4	2.3	0.7	7.2	.15
	≥2 y old	103		38.6	1.0			
Aboriginal/Torres Strait Islander	Yes	25	267	9.4	0.6	0.1	4.3	.57
Weight for age	≥95th centile	23	267	8.6	2.2	0.6	8.3	.25
	85–94th centile	26		9.7	0.6	0.1	4.6	.61
	<85% centile	218		81.7	1.0			
Co‐infection	Viral/bacterial	107	267	40.2	1.2	0.5	3.2	.70
Hospital episode number	>1	179	267	67.0	0.8	0.2	2.0	.58
	1	88		33.0	1.0			
Influenza strain	Influenza B	54	265	20.4	2.7	1.0	7.4	.05
	Influenza A	211		79.6	1.0			
Influenza A subtype	Influenza A non‐H1N1	96	211	45.5	1.5	0.4	5.0	.54
	Influenza A H1N1	115		54.5	1.0			
Comorbidities b	Yes	126	267	47.2	1.8	0.7	4.9	.23
Chronic lung disease	Yes	65	267	24.3	1.3	0.5	4.1	.55
Cardiac abnormality	Yes	20	267	7.5	2.7	0.7	10.4	.14
Renal disease	Yes	7	267	2.6	2.4	0.3	20.2	.43
Immunocompromised^c^	Yes	15	267	5.6	–	–	–	–
Iron deficiency anaemia	Yes	18	267	6.7	4.2	1.4	12.9	.01
Neurological disorder	Yes	39	267	14.6	2.4	0.8	7.3	.11
Asthma	Yes	42	267	15.7	1.1	0.3	3.9	.91
Atopic history^a^	Yes	66	267	24.7	1.2	0.4	3.5	.76
Food allergy	Yes	8	267	3.0	5.1	1.0	27.1	.06

a Atopic history includes asthma, atopic dermatitis and hay fever.

b As defined in the AIH.^14^

c Unable to fit model as no patients in this category required high‐level care.

In a multivariable model for predictors of influenza infection requiring high‐level care (HDU/ICU), influenza type (B strain), iron deficiency and food allergy were shown to be statistically significant independent predictors of influenza severity (OR 3.7, 95% CI 1.3–10.9; *P *= .02, OR 4.8, 95% CI 1.4–16.1; *P *= .01 and OR 9.7, 95% CI 1.5–61.4; *P *= .02, respectively) (Table 4).

**Table 4:** Final multivariable logistic regression model

Severity	Level	Odds ratio	95% CI	*P* value
Influenza strain	Influenza B	3.7	1.3, 10.9	.018
	Influenza A	1.00		
Iron deficiency anaemia	Yes	4.8	1.4, 16.1	.011
	No	1.0		
Food allergies	Yes	9.7	1.5, 61.4	.016
	No	1.0		
Age category	<2 y	2.8	0.8, 9.1	.097
	≥2 y	1.0		
Presence of AIH risk factor^14^	Yes	2.3	0.8, 6.6	.124
	No	1.0		

As the majority of severe cases (14/18, 78%) and iron deficiency cases (19/26, 73%) were aged less than 2 years, we repeated the multivariable model restricting to children less than 2 years of age to further assess the association with severity. Despite the reduced sample size (*n* = 163), iron deficiency remained an independent predictor of severity (OR 4.3, 95% CI 1.1–16.3; *P *= .03) within this age group.

## 4 Discussion

This study assessed the disease burden and the impact of various risk factors on the severity of influenza in children under 5 years of age including the impact of premature birth, low birthweight, age <2, obesity, indigenous ethnicity, infecting influenza strain, co‐infections, use of antivirals, pre‐existing medical comorbidities and other potential risk factors. Influenza can cause a severe disease in young children, even in those without underlying medical risk factors. Of the risk factors studied, influenza type B, iron deficiency anaemia and food allergy were identified as independent risk factors for severe influenza disease in children under five.

Adjusting for influenza strain, age category, food allergy and presence of a medical risk factor for severe disease (as per the Australian Immunisation Handbook^19^), children with iron deficiency anaemia were nearly five times more likely to be admitted to high‐level care. Iron deficiency anaemia was an important predictor of disease severity particularly in the under 2‐year age group. This is relevant as children under 2 years are also a higher risk age group for iron deficiency due to factors such as inadequate intake and amplified iron requirements for rapid growth.^20^ In previous literature, iron deficiency has been reported as a comorbidity associated with influenza A.^21^ In addition, anaemia has been identified as a risk factor for respiratory failure in children admitted to hospital for community‐acquired pneumonia.^22^ Iron has many important functions in the body including regulation of the immune system,^23^, ^24^ and therefore, deficiency could compromise immune function and impact on acquisition and severity of infections including influenza.

The presence of a food allergy was identified as a risk factor for increased severity of influenza. Our results suggest that children with reported food allergies are over 9 times more likely to require high‐level care for influenza infection. Our results need to be interpreted with caution as the sample size of this group was small. There is little literature available on food allergies and severity of infections. Ciprandi et al.^25^ provided evidence that allergic children had more numerous and severe respiratory infections than non‐allergic children.^25^ A study by Silverberg showed that children with atopic dermatitis and warts had higher rates of extracutaneous infections, including influenza and pneumonia, suggesting that the immune dysfunction involved in the development of atopic disease and warts may also play a role in the increased risk of infections.^26^ Higher rates of food allergy were also demonstrated in children with atopic dermatitis and warts in their study. However, to our knowledge, there is no published literature linking food allergies and an increased risk of severity of respiratory infections. An atopic history in our cohort of hospitalised children was not a significant predictor of severe influenza, potentially due to the small sample size. Allergic individuals display immunological mechanisms that are characterised by Th‐2 polarisation, hence reducing Th‐1 responses required for fighting respiratory infections.^27^ Further investigation is required to determine whether this is a true association, as food allergies such as egg allergy may impact on acceptance of influenza vaccination.

There was no significant association identified between different influenza A strains (H1N1 vs not H1N1) and severity; however, influenza B was found to be significantly associated with more severe infection compared with Influenza A. Children were over three times more likely to be admitted to ICU or HDU if infected with influenza B (OR 3.7, CI 1.3–10.9 and *P *= .02). Severe disease and fatalities with influenza B infection in children have also been reported by Glezen et al.^28^ and Paddock et al,^29^ who reported no recognisable risk factor for fatal influenza B infection in their series other than young age (<18 years). However, there have been other conflicting studies that have found influenza A to cause more severe illness than influenza B.^10^, ^30^ Severity may depend on the circulating and infecting influenza B lineage.

The predominant strains of influenza have changed over the last 5 years indicating variability in circulation. A decrease in circulating A(H1N1) in 2012 may have been due to the increased vaccination rate during the 2009 pandemic and high exposure to natural infection during 2009–2011 with resulting herd immunity. Vaccine mismatch may have also contributed to the increase in influenza B admissions. Since the 1980s, the antigenically distinct B lineages, B/Victoria and B/Yamagata have co‐circulated in various countries, including Australia and the Asia–Pacific region, but the proportion of each lineage varies from year to year and from country to country.^31^ Predicting the predominant circulating influenza B lineage has been difficult, but is an important consideration for influenza vaccine formulation decisions, as only one influenza B component is traditionally included in the annual trivalent influenza vaccine. In 2015, in addition to the government‐funded trivalent vaccine, a licenced quadrivalent influenza vaccine has also been made available for the first time in Australia on the private market and included in the funded national immunisation programme (NIP) for 2016. This may provide the opportunity to reduce the risk of vaccine mismatch and minimise the severity of influenza infection.

In Australia, influenza immunisation is recommended for all persons 6 months and older who wish to receive protection against influenza. Children aged 6 months to 5 years are eligible for free influenza vaccine in Western Australia but not in other states or as part of the NIP unless the child is of Aboriginal or Torres Strait Islander origin, or has underlying medical conditions placing them at risk of severe influenza.^19^ A high proportion of children in this study had medical conditions that would have enabled them to receive free influenza vaccination; however, only four children had documented evidence of influenza vaccination during the year prior to admission. This may be an underestimation of the true vaccination coverage as, unlike routine childhood vaccines, influenza vaccination is not routinely recorded on the Australian Childhood Immunisation Register. However, previous Australian studies suggest that the uptake of influenza vaccine among Australian children is low, even in at risk groups for whom influenza vaccination is funded.^32^ Low vaccination rates in these children might be a result of parents' hesitancy to vaccinate against influenza, due to poor knowledge about the vaccine or concern about side effects or lack of immunisation provider recommendations,^33^ all of which are potential avenues for further exploration.

Another issue, not explored in this study, is the impact of host genetic factors on influenza disease susceptibility and/or severity. Genetic polymorphisms in certain immune effector proteins such as the interferon‐induced transmembrane protein‐3 (IFITM3)^34^ and the tumour necrosis factor‐alpha (TNF‐α) and interleukin genes^35^ have been reported in the literature as being linked to poorer outcomes with influenza. An IFITM3 genetic variant, rs12252‐C, which is more prevalent among East Asian populations, has been shown to increase influenza susceptibility and disease severity.^36^ Nosocomial influenza infections, whilst infrequent, were also identified as an important group impacting on influenza disease burden as the majority required high‐level care.

Our results may be subject to bias due to the small number of cases requiring high‐level care, which is one of the major limitations of our study. This was a retrospective study, which limited information resources and is potentially subject to bias and conducted in a tertiary care hospital where more severe cases may be transferred. Nevertheless, further investigation of the association between increased disease severity in children with food allergies and iron deficiency anaemia should be considered. New quadrivalent vaccines containing two influenza B strains in addition to two influenza A strains may provide additional protection against severe Influenza B‐related disease.

In summary, the associations observed between disease severity and influenza type B, iron deficiency anaemia and food allergies are newly identified factors that affect influenza severity in South Australian children under 5 years of age. These findings have important implications for preventive strategies and warrant further study. Confirmed iron deficiency anaemia or food allergy could be included as additional conditions which meet eligibility for government‐funded influenza vaccination. Furthermore, the association between Influenza B and increased disease severity support the recommendation of quadrivalent influenza vaccine for children.

## Conflict of Interest and Source of Funding

There are no conflict of interests to declare by the authors that are directly related to this manuscript. However, HM, MC and SM's institution has received funding for investigator‐led research from bioCSL, Novartis and GSK.

## Acknowledgement

Helen Marshall is supported by the National Health Medical Research Council Career Development Fellowship (1084951).
